# Characterization of 29 newly isolated bacteriophages as a potential therapeutic agent against IMP-6-producing *Klebsiella pneumoniae* from clinical specimens

**DOI:** 10.1128/spectrum.04761-22

**Published:** 2023-09-19

**Authors:** Kohei Kondo, Satoshi Nakano, Junzo Hisatsune, Yo Sugawara, Michiyo Kataoka, Shizuo Kayama, Motoyuki Sugai, Mitsuoki Kawano

**Affiliations:** 1 Antimicrobial Resistance Research Center, National Institute of Infectious Diseases, Higashimurayama, Tokyo, Japan; 2 Department of Pathology, National Institute of Infectious Diseases, Toyama, Shinjuku-ku, Tokyo, Japan; 3 Department of Nutritional Sciences, Nakamura Gakuen University, Jonan-Ku, Fukuoka, Japan; University of Pittsburgh School of Medicine, Pittsburgh, Pennsylvania, USA

**Keywords:** bacteriophages, *Klebsiella pneumoniae*, *bla*_IMP-6_, trade-off, phage-resistant bacteria, phage library

## Abstract

Carbapenemase-producing *Enterobacteriaceae* (CPE) are one of the most detrimental species of antibiotic-resistant bacteria globally. Phage therapy has emerged as an effective strategy for the treatment of CPE infections. In western Japan, the rise of *Klebsiella pneumoniae* strains harboring the pKPI-6 plasmid encoding *bla*_IMP-6_ is of increasing concern. To address this challenge, we isolated 29 phages from Japanese sewage, specifically targeting 31 *K*. *pneumoniae* strains and one *Escherichia coli* strain harboring the pKPI-6 plasmid. Electron microscopy analysis revealed that among the 29 isolated phages, 21 (72.4%), 5 (17.2%), and 3 (10.3%) phages belonged to myovirus, siphovirus, and podovirus morphotypes, respectively. Host range analysis showed that 18 *Slopekvirus* strains within the isolated phages infected 25–26 *K*. *pneumoniae* strains, indicating that most of the isolated phages have a broad host range. Notably, *K. pneumoniae* strain Kp21 was exclusively susceptible to phage øKp_21, whereas Kp22 exhibited susceptibility to over 20 phages. Upon administering a phage cocktail composed of 10 phages, we observed delayed emergence of phage-resistant bacteria in Kp21 but not in Kp22. Intriguingly, phage-resistant Kp21 exhibited heightened sensitivity to other bacteriophages, indicating a “trade-off” for resistance to phage øKp_21. Our proposed phage set has an adequate number of phages to combat the *K. pneumoniae* strain prevalent in Japan, underscoring the potential of a well-designed phage cocktail in mitigating the occurrence of phage-resistant bacteria.

**IMPORTANCE:**

The emergence of *Klebsiella pneumoniae* harboring the *bla*_IMP-6_ plasmid poses an escalating threat in Japan. In this study, we found 29 newly isolated bacteriophages that infect *K. pneumoniae* strains carrying the pKPI-6 plasmid from clinical settings in western Japan. Our phages exhibited a broad host range. We applied a phage cocktail treatment composed of 10 phages against two host strains, Kp21 and Kp22, which displayed varying phage susceptibility patterns. Although the phage cocktail delayed the emergence of phage-resistant Kp21, it was unable to hinder the emergence of phage-resistant Kp22. Moreover, the phage-resistant Kp21 became sensitive to other phages that were originally non-infective to the wild-type Kp21 strains. Our study highlights the potential of a well-tailored phage cocktail in reducing the occurrence of phage-resistant bacteria.

## INTRODUCTION

Carbapenemase-producing *Enterobacteriaceae* (CPE) pose a considerable risk in clinical settings worldwide. Among them, *Klebsiella pneumoniae*, a member of the *Enterobacteriaceae* family, is a leading cause of nosocomial infections and a major contributor to life-threatening infections caused by multidrug-resistant bacteria globally (1). The *bla*_IMP_ genes belong to the class B metallo-β-lactamases. *bla*_IMP-1_ and *bla*_IMP-6_ genes are predominantly identified in CPE isolated in Japan (2, 3), whereas other types of carbapenemases such as NDM, KPC, and OXA-48 are more prevalent in CPE strains isolated from other countries (4). *Klebsiella pneumoniae* strains carrying the *bla*_IMP-6_-encoding pKPI-6 plasmid (5), which is susceptible to imipenem but resistant to meropenem, have become increasingly common in clinical settings in western Japan since their emergence in 2009 (6). These strains are therefore of major concern in clinical settings because of their inappropriate response to commonly used antibiotics.

Recently, bacteriophage therapy has gained considerable attention as an alternative treatment for infections caused by antimicrobial-resistant bacteria (7). Phage therapy targeting *Staphylococcus aureus* (8) and *Mycobacterium tuberculosis* (9) has been administered successfully to patients. Furthermore, a recent study has demonstrated the efficacy of phage therapy against CPE in clinical settings (10). Thus, phage therapy is now recognized as a highly reliable strategy to combat nosocomial pathogens.

However, the use of phages against bacteria has led to the emergence of phage-resistant bacteria (11) *in vitro* (12) and *in vivo* (13). To address this issue, phage cocktails, which consist of multiple phage types, are often used to prevent the emergence of phage-resistant bacteria. Establishing a phage bank is pertinent for the rapid deployment of phage cocktails in clinical settings (14), especially in emergency cases. As national phage banks are pertinent for the instant management of contingent nosocomial pathogen outbreaks, several countries have constructed public phage banks for the efficient use of phage therapy (14, 15). However, in Japan, there is currently no public phage bank optimized to address the evolving trends in antibiotic-resistant bacteria.

In this study, we isolated and characterized 29 bacteriophages targeting IMP-6-producing *K. pneumoniae* and *Escherichia coli* clinical isolates as the first step in establishing a public phage library in Japan. Additionally, we described the mechanisms by which phage cocktails can effectively reduce the emergence of phage-resistant *K. pneumoniae* strains.

## RESULTS

### Phage hunting and morphological analysis of newly isolated bacteriophages

**Fig 1:**
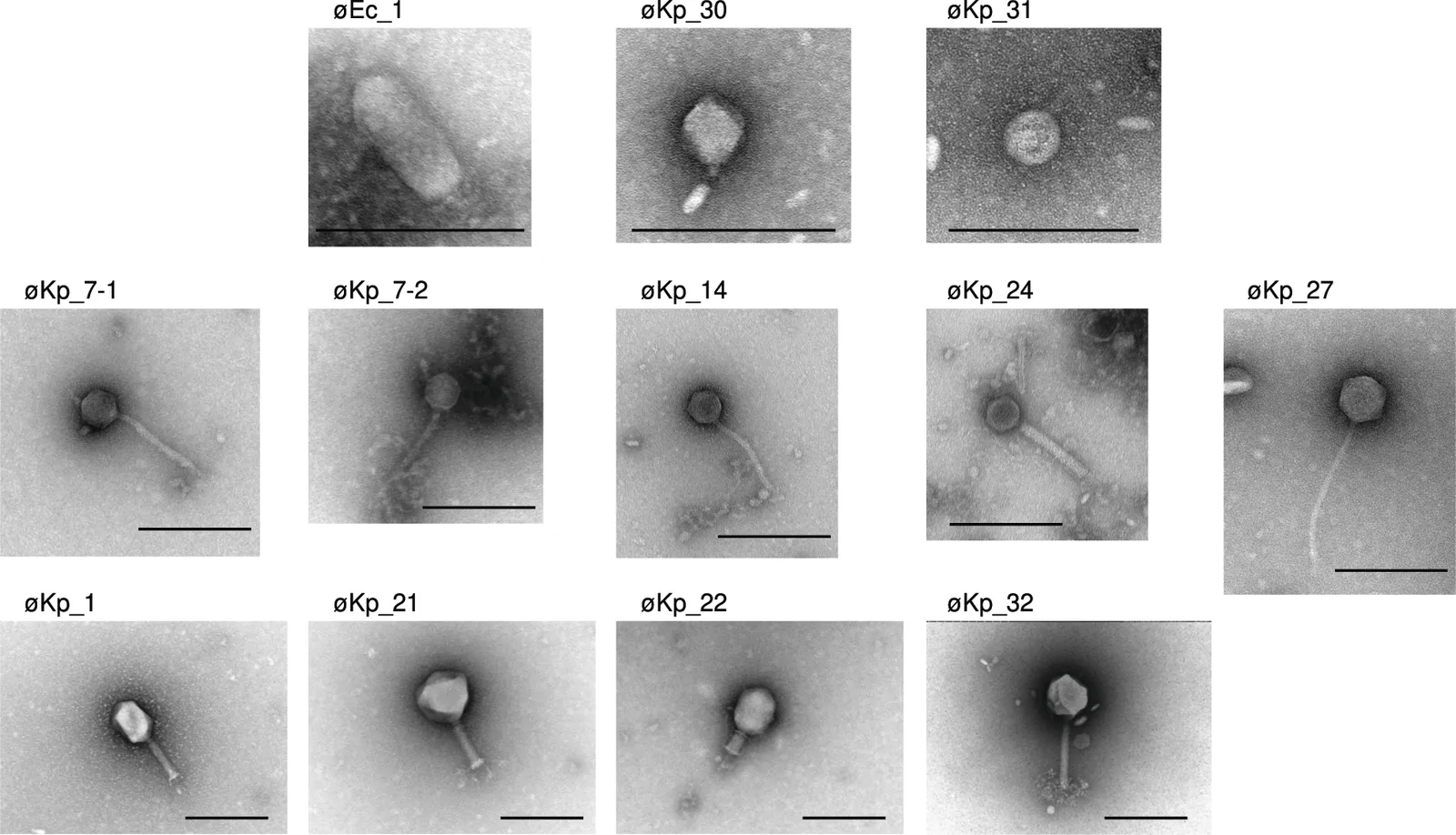
Transmission electron microscopy images of 29 isolated phages. Each sample was negatively stained and magnified at ×50,000. Representative myovirus morphotype phages, øKp_1, øKp_21, øKp_22, and øKp_32, are displayed. All podovirus morphotype phages and siphovirus morphotype phages are shown. The scale bar in each image represents 200 nm.

We performed a phage hunting expedition in the sewage system of western Japan, resulting in the isolation of 29 phages targeting 32 strains of *K. pneumoniae* (Kp1–Kp32) and one strain of *E. coli* (Ec1), all harboring the pKPI-6 plasmid. It is worth noting that 81.2% (26/32) of the hosts used in this study belonged to sequence type 37, which represents the predominant clone frequently encountered in Japanese clinical settings and commonly associated with the presence of IMP-6 (Table S1) (5). Each phage was assigned a name corresponding to its host number. For instance, the phages øEc_1 and øKp_1 were isolated from *E. coli* strains Ec1 and *K. pneumoniae* strain Kp1, respectively, as their corresponding hosts. All phage-corresponding host combinations are listed in Table S2. We did not identify suitable phages capable of infecting *K. pneumoniae* strains (Kp2, Kp6, Kp25, Kp28, and Kp29). Morphological analysis using electron microscopy revealed that 21 out of 29 (72.4%) isolated phages belonged to the myovirus morphotype, 5 out of 29 (17.2%) belonged to the siphovirus morphotype, and 3 out of 29 (10.3%) belonged to the podovirus morphotype (Fig. 1). All transmission electron microscopy (TEM) images of the myovirus morphotype are shown in Fig. S1 except representative phages (øKp_1, øKp_21, øKp_22, and øKp_32) which are shown in Fig. 1. Notably, based on transmission electron microscopy images, phage øEc_1 was identified as belonging to the podovirus morphotype and C3 morphotype (honeycomb-like) phages (16 – 18), characterized by an elongated head (height, 136.6 nm ± 1.8 nm; width, 61.7 nm ± 3.6 nm; tail, 15.8 nm ± 2.3 nm) (Fig. 1). øEc_1 formed turbid plaques on the *E. coli* Ec1 strain. øKp_21 was classified as a myovirus morphotype (height, 133.8nm± 3.1 nm; width, 137.1 nm ± 1.1 nm; tail, 109.7 nm ± 1.0 nm) (Fig. 1). Furthermore, øKp_21 possessed a branched tail (tail spike) fiber and formed clear plaques on a lawn of *K. pneumoniae* Kp21.

### Bacteriophage classification

**Fig 2:**
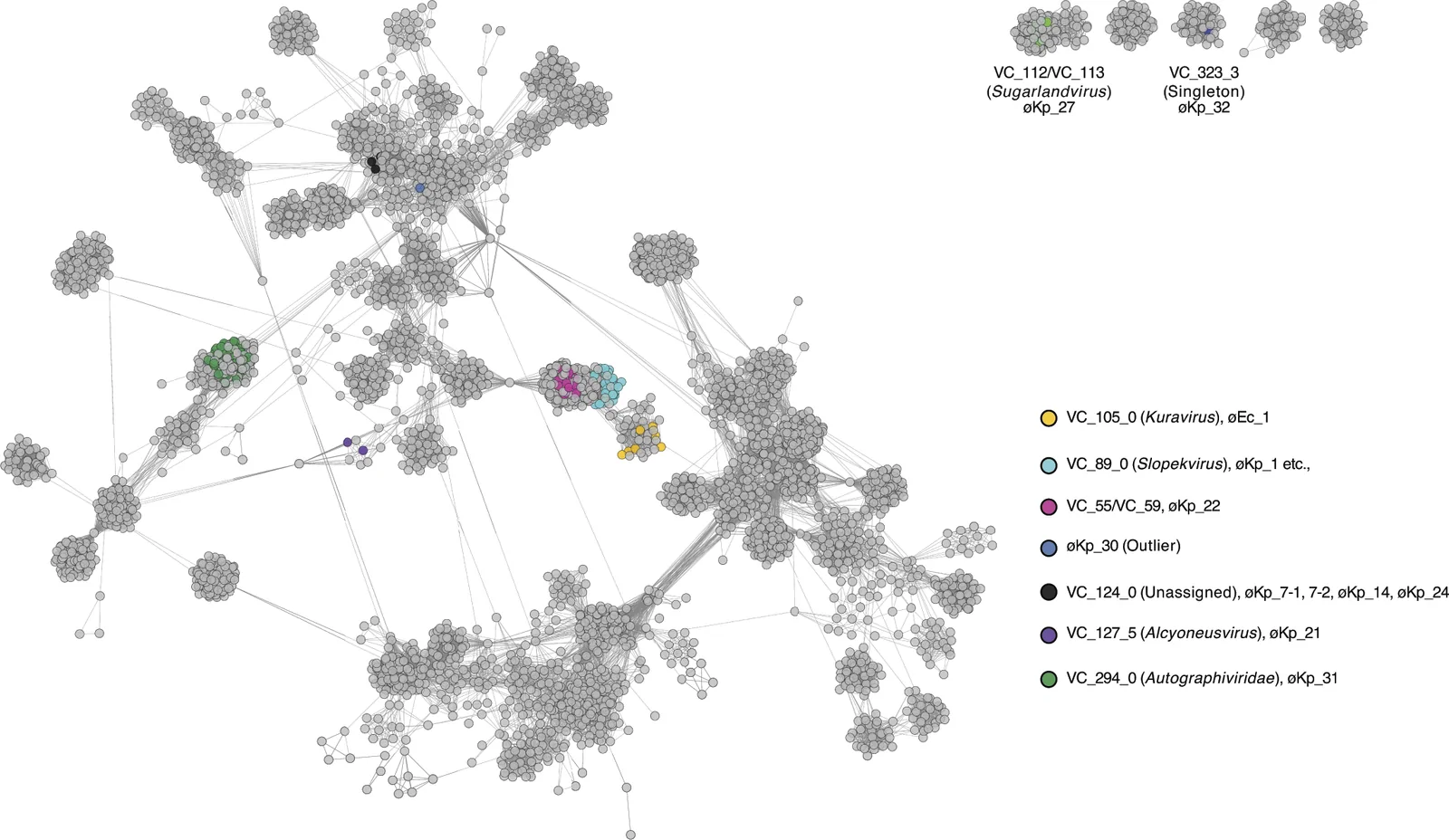
Network images of the newly isolated phages based on shared protein ortholog families. vConTACT2 v0.11.3 was used for clustering each phage. vConTACT2 assigned viral cluster (VC) to each phage. Our phages were divided into nine VCs. Each VC, including our phages, is highlighted in different colors.

To classify the newly isolated phages, we employed a two-step approach. First, we conducted phage classification using network analysis based on phage-encoded protein ortholog families using vConTACT2 (19). Subsequently, we performed an average nucleotide identity (ANI) analysis. vConTACT2 generated “viral clusters” based on shared protein similarities and proposed taxonomic classifications for the phages of interest. vConTACT2 integrated our isolated phages into nine viral clusters (VC_105_0, VC_89_0, VC_55/VC_59, VC_124_0, VC127_5, VC_294_0, VC_112/VC_113, VC_323_3, and an outlier) (Fig. 2).

Among the three phages exhibiting a podovirus morphotype, øEc_1 and øKp_31 were classified as *Kuravirus* at the genus level and *Autographiviridae* at the family level, respectively (Fig. 2). Conversely, øKp_30 could not be classified at either the family or genus level using vConTACT2 (Fig. 2). øEc_1 exhibited nucleotide identities of 97.9% and 95.4% with *Escherichia* phage MN03 (NC_070990.1) and øEco32, respectively (16) (Fig. S2A), both classified as *Kuravirus*. øKp_31 displayed nucleotide identities of 95.7% and 93.4% with KPP-5 (20) and T3 phage (NC_003298), respectively. For øKp_30, nucleotide identities of 95.3% and 95.9% were observed with *Escherichia* phage CUS-3 (21) and vB_EcoP_Kapi1 (22), both classified as *Lederbergvirus* in the National Center for Biotechnology Information (NCBI) taxonomy database (Fig. S2A). This indicates that øKp_30 belongs to the *Lederbergvirus*. It is worth noting that *Lederbergvirus* mainly infects *Escherichia coli* or *Salmonella* species, and we did not find any *Lederbergvirus* infecting *K. pneumoniae* in the NCBI database. Therefore, this may be the first report of a complete genome of a *Lederbergvirus* infecting *K. pneumoniae*. Life cycle analysis *in silico* suggests that øKp_30 is likely a temperate phage (Table S2), but we have not confirmed whether øKp_30 integrates into the host chromosome.

Among the five phages exhibiting a siphovirus morphotype, øKp_27 was classified as *Sugarlandvirus*, while the remaining four siphovirus morphotype phages (øKp_7–1, øKp_7–2, øKp_14, and øKp_24) were clustered in the same viral cluster (VC_124_0) and were not classified at the family or genus levels based on vConTACT2 (Fig. 2). These unclassified siphoviruses displayed similar nucleotide identities to each other (Fig. S3) and were highly similar to vB_Kp3 (ON602766) and vB_KleS-HSE3 (23), which are also unclassified phages (Fig. S2B). øKp_27 exhibited nucleotide identities of 95.2% and 95.3% with Sugarland phages (24) and vB_Kpn_IME260 phage (25) (Fig. S2B).

Of the 21 phages exhibiting a myovirus morphotype, øKp_21 is classified as *Alcyoneusvirus*, whereas øKp_22 and øKp_32 could not be classified at the family or genus levels (genus not assigned) based on vConTACT2. All other myovirus phages are classified under the *Slopekvirus* genus. The *Slopekvirus* phages isolated in this study share a remarkably high sequence identity (over 99.8%) and coverage (over 98.7%) with each other, suggesting they are variants of same species. øKp_1, classified as a *Slopekvirus,* exhibits nucleotide identities of 98.8% and 98.4% with *Klebsiella* phage PMBT1 (26) and Miro (27) (Fig. S2C), respectively. Among the other myovirus morphotype phages, øKp_21 exhibits 97.6% nucleotide identity with *Klebsiella* phage Muenster (28). øKp_22 displays 97.9% nucleotide identity with *Klebsiella* phage KP1 (29), classified as *Jiaodavirus* (Fig. S2C) in the NCBI taxonomic database. øKp_32 exhibits 95.9% nucleotide identity with *Klebsiella* phage Miami (30) (Fig. S2C). øKp_21 and øKp_32 possess genome sizes of 353,382 bp (532 CDS) and 251,460 bp (275 CDS), respectively, indicating their classification as jumbo phages (Table S2).

### Other genomic information

Recently, phage defense systems and anti-phage defense mechanisms have garnered considerable attention due to their impact on infection efficacy and host range, which are crucial for the successful implementation of phage therapy. Anti-CRISPR (Acr) proteins, which protect phages from the host CRISPR immune system, represent well-characterized anti-defense systems (31). We predicted Acr and Acr-associated regulator (Aca) proteins from our representative phage genomes using AcrFinder (Table S3). The results revealed the formation of genomic clusters of Acr and Aca, consistent with previous reports (32), and majority of phages in our study possess at least one Acr clustered genomic island. Notably, we identified 18 predicted Acr and/or Aca proteins encoded by øKp_27 and 25 by øKp_32. Most of these proteins were found to be less than 200 amino acids long.

To determine the number of encoded tRNAs, we employed tRNAscan-SE (Table S2). Remarkably, øKp_22 and øKp_27 encode 16 and 27 tRNAs, respectively, forming tRNA islands (Fig. S4; Table S4).

### Host range determination and analysis of the correlation between plaque size and efficiency of plating

**Fig 3:**
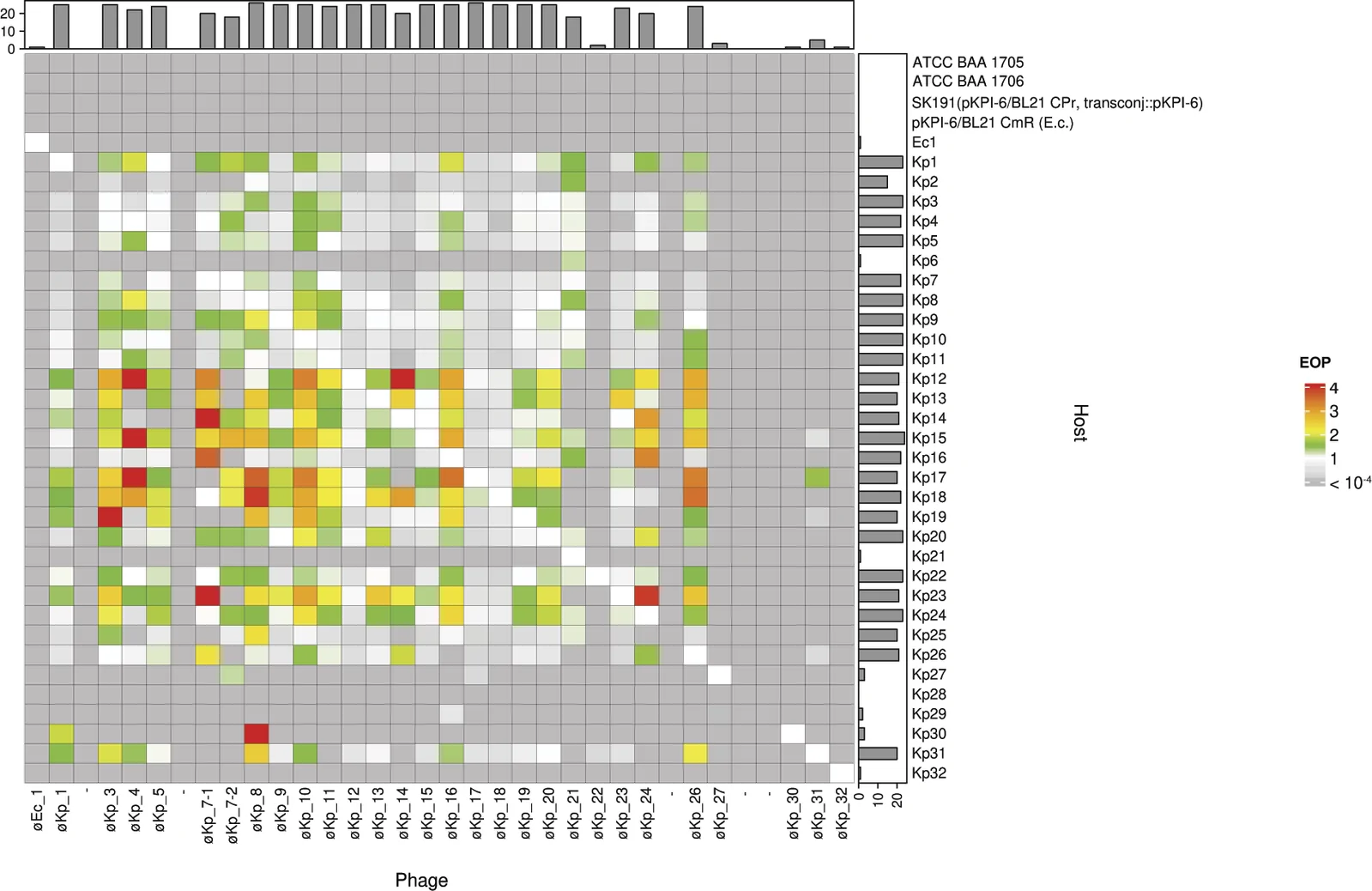
A heatmap illustrating the host range of each phage. The X- and Y-axes represent phages and host strains, respectively. *Klebsiella pneumoniae* ATCC BAA 1705 and ATCC BAA 1706 served as standard strains, whereas *Escherichia coli* SK191 and BL21 served as control strains. The color in the heatmap represents the EOP. Bar charts on the X- and Y-axes represent the number of infections in each phage and host, respectively.

We next determined the host range and its efficiency of plating (EOP) for all phage-host combinations (Fig. 3) (Table S5). We selected two standard strains (ATCC BAA 1705 and ATCC BAA 1706) as the controls for *K. pneumoniae*. Although no newly isolated phages against Kp2, Kp6, Kp25, Kp28, or Kp29 were isolated, the host range experiment indicated that several isolated phages formed plaques on Kp2, Kp6, Kp25, and Kp29 but not Kp28 (Fig. 3). Phages øKp_8 and øKp_17 exhibited the broadest host range, infecting 26 out of 32 *K*. *pneumoniae* host strains. Most phages (øKp_3, øKp_5, øKp_9, øKp_10, øKp_12, øKp_13, øKp_15, øKp_16, øKp_18, øKp_19, øKp_20, and øKp_26) demonstrated plaque formation on 25 host *K. pneumoniae* strains, indicating that these phages have a broad host range (infecting ≥25 host strains). Conversely, several phages exhibited a narrow host range (infecting ≤4 host strains). For instance, øKp_31 infected Kp15, Kp17, Kp26, and Kp31. øKp_27 (*Sugarlandvirus*), øKp_30 (*Lederbergvirus*), and øKp_32 (myovirus) infected only Kp27, Kp30, and Kp32, respectively (Fig. 3). Overall, our phage set encompasses *K. pneumoniae* commonly encountered in clinical settings and offers an adequate variety of phage types to facilitate the development of a phage cocktail targeting *K. pneumoniae* in this study.

We observed a positive correlation between EOP values and the formation of large plaques. To investigate this further, we conducted a correlation analysis between EOP values and plaque size for eight representative phages that exhibited distinct host range patterns. Pearson’s correlation coefficients (*R* values) were 0.75 for øKp_1, 0.86 for øKp_7–1, 0.70 for 7–2, 0.55 for øKp_14, 0.25 for øKp_21, 0.80 for øKp_24, 0.83 for øKp_27, and 0.65 for øKp_31 (Fig. S5). These results demonstrate a positive association between plaque size and EOP. We hypothesized that EOP and plaque size are determined by adsorption efficiency, phage life cycle time, and/or burst size. To assess the factors influencing plaque size in our phages, we conducted adsorption analysis and one-step growth curve experiments on three different phages: myovirus øKp_1 (*Slopekvirus*), siphovirus øKp_7–1 (genus not assigned), and podovirus øKp_31 (*Autographiviridae*) (Fig. S6). We selected two hosts with lower and higher EOP values for each phage. The adsorption assay revealed no statistically significant difference between the two hosts for all phages (*P* = 0.117 for øKp_1, *P* = 0.351 for øKp_7–1, and *P* = 0.127 for øKp_31) (Fig. S6). However, the host with a higher EOP exhibited a significantly larger burst size compared to the host with a lower EOP at 38 min for øKp_1, 40 min for øKp_7–1, and 40 min for øKp_31 (*P* = 0.0229, 0.0493, and 0.00414, respectively). These findings suggest that plaque size for each host is primarily defined by burst size rather than adsorption efficiency.

### OD_600_ kinetics of *K. pneumoniae* challenged with phages

**Fig 4:**
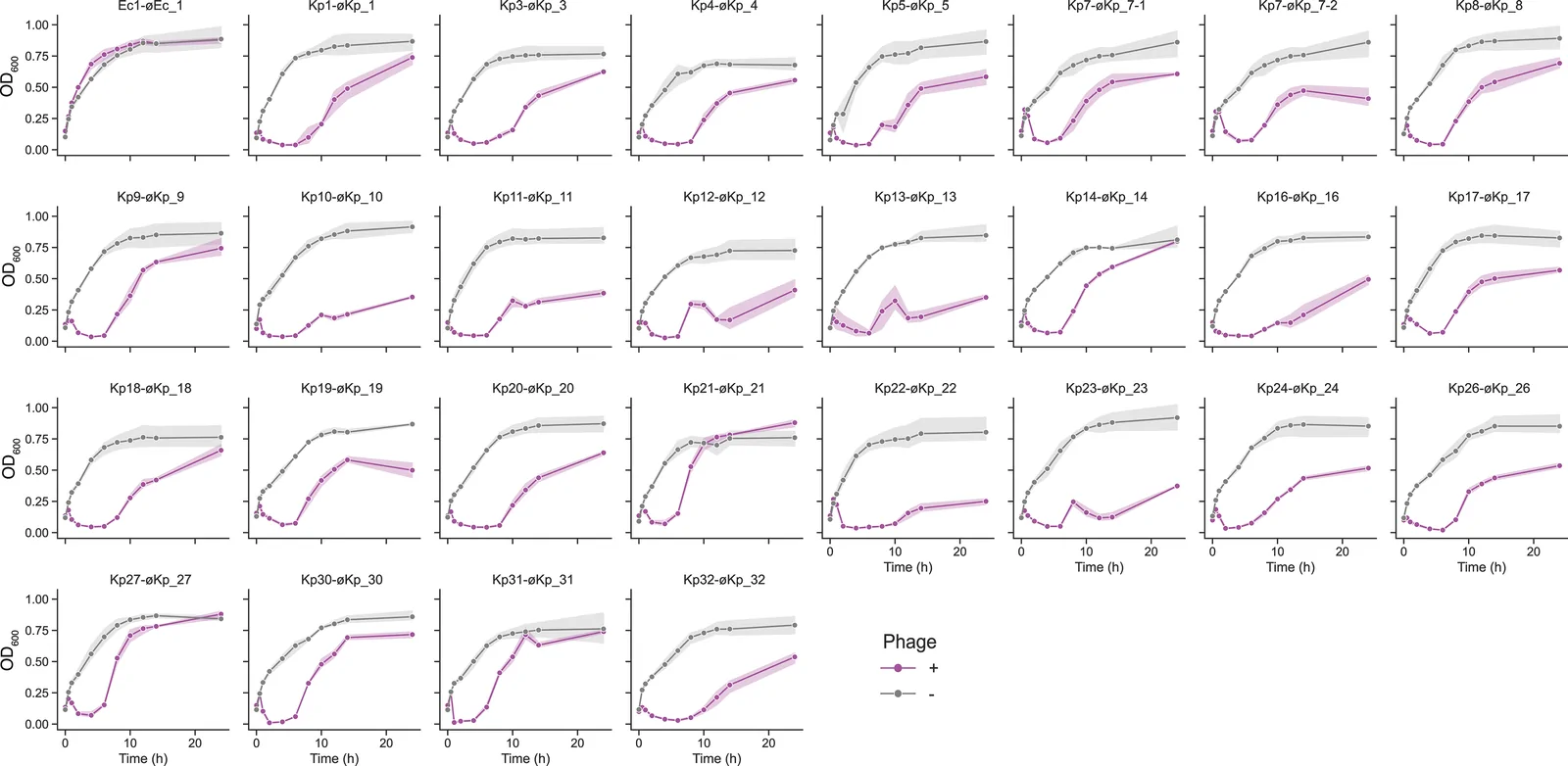
OD_600_ kinetics of indicator bacteria incubated with phages. Bacterial strains were incubated until reaching an OD_600_ of 0.1, after which each phage was added at a concentration of 10^9^ pfu/mL. OD_600_ measurements were taken at appropriate time intervals for up to 24 h. All experiments in this section were performed in triplicate. Gray and magenta represent the OD_600_ without (negative control) and with phages, respectively.

The OD_600_ kinetics were analyzed for all phage-indicator host combinations. Individual phages were added at a concentration of 10^8^ pfu/mL (multiplicity of infection [MOI)],~10) when the host bacteria entered the exponential phase (OD_600_ = 0.1). Within 1 h of phage addition, a noticeable decrease in OD_600_ was observed, while the mixture of Ec1 and øEc_1 did not exhibit a decrease and maintained an OD_600_ level similar to that of the host strain without any phages (Fig. 4). Although øEc_1 and øKp_30 were predicted to be temperate phages (Table S2), the OD_600_ of øKp_30 decreased 1 h after its addition, resembling the OD_600_ kinetics of other virulent phages. Therefore, øKp_30 has the potential to be applied in phage therapy. Furthermore, the OD_600_ in all phage combinations began to rise again 6–10 h after the addition of each phage. These findings indicate the emergence of phage-resistant bacteria in almost all phage-host combinations.

### Cocktail analysis of phage-resistant bacteria Kp21 and Kp22

**Fig 5:**
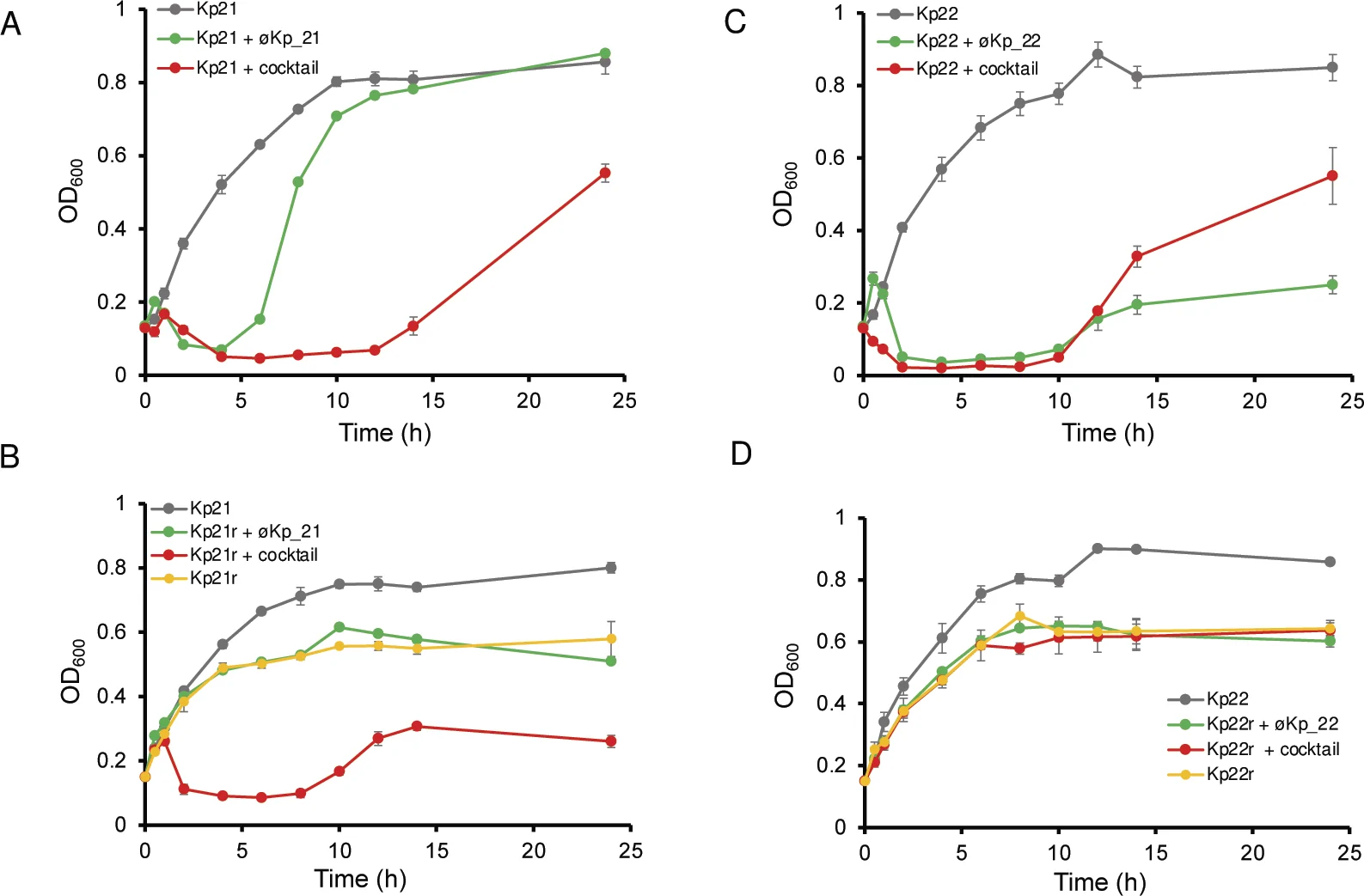
Cocktail experiment of Kp21r with Kp22r. Cocktail experiment of Kp21r with Kp22r. OD_600_ kinetics of Kp21 and Kp21r combined with phage cocktail are shown in panels A and B, respectively. OD_600_ kinetics of Kp22 and Kp22r combined with phage cocktail are shown in panels C and D, respectively. Phage-resistant Kp21 (Kp21r) and Kp22 (Kp22r) strains were obtained from the culture medium after 24 h of incubation with øKp_21 or øKp_22. The cocktail comprised 10 phages, each at a concentration of 10^7^ pfu/mL. OD_600_ measurements were taken at appropriate time intervals for up to 24 h. All experiments in this section were performed in triplicate.

Kp21 exhibited susceptibility only to øKp_21, whereas the Kp22 strain was susceptible to 23 different phages (Fig. 3). Notably, øKp_21 could infect 18 hosts, including Kp21, whereas øKp_22 could only infect Kp22 and Kp31 strains (Fig. 3). Given this contrast, we selected øKp_21 and øKp_22 for the phage cocktail experiment. The phage cocktail comprised 10 phages (øKp_16–26), encompassing eight *Slopekviruses*, one *Alcyoneusvirus*, and one siphovirus. In the Kp21 and øKp_21 combination, the OD_600_ increased after 6 h and eventually reached the level of the negative control after 24 h (Fig. 5A). However, the OD_600_ did not increase until 14 h after Kp21 was combined with the phage cocktail (Fig. 5A). This finding suggests that the phage cocktail effectively delayed the emergence of phage-resistant Kp21 in the *in vitro* assay. Conversely, the OD_600_ of the cocktail against Kp22 exhibited a similar pattern to that of single øKp_22, increasing again after 10 h (Fig. 5C). Thus, the phage cocktail failed to delay the emergence of phage-resistant Kp22. We isolated øKp_21-resistant Kp21 (Kp21r) and øKp_22-resistant Kp22 (Kp22r) using the methodology described in the Materials and Methods section. Although the OD_600_ kinetics in the mixture of Kp21r and øKp_21 did not decrease and approximated those of Kp21r without any phages, the OD_600_ was reduced in the Kp21r-phage cocktail combination (Fig. 5B). This result indicates that the Kp21r strain is resistant to phage 21 but susceptible to the phage cocktail. However, the OD_600_ did not decrease in either the Kp22r-øKp_22 or Kp22r-cocktail combinations, suggesting that the phage-resistant Kp22r is not susceptible to either øKp_22 or the phage cocktail (Fig. 5D).

### Phage sensitivity shift between Kp21 and Kp21r

**Fig 6:**
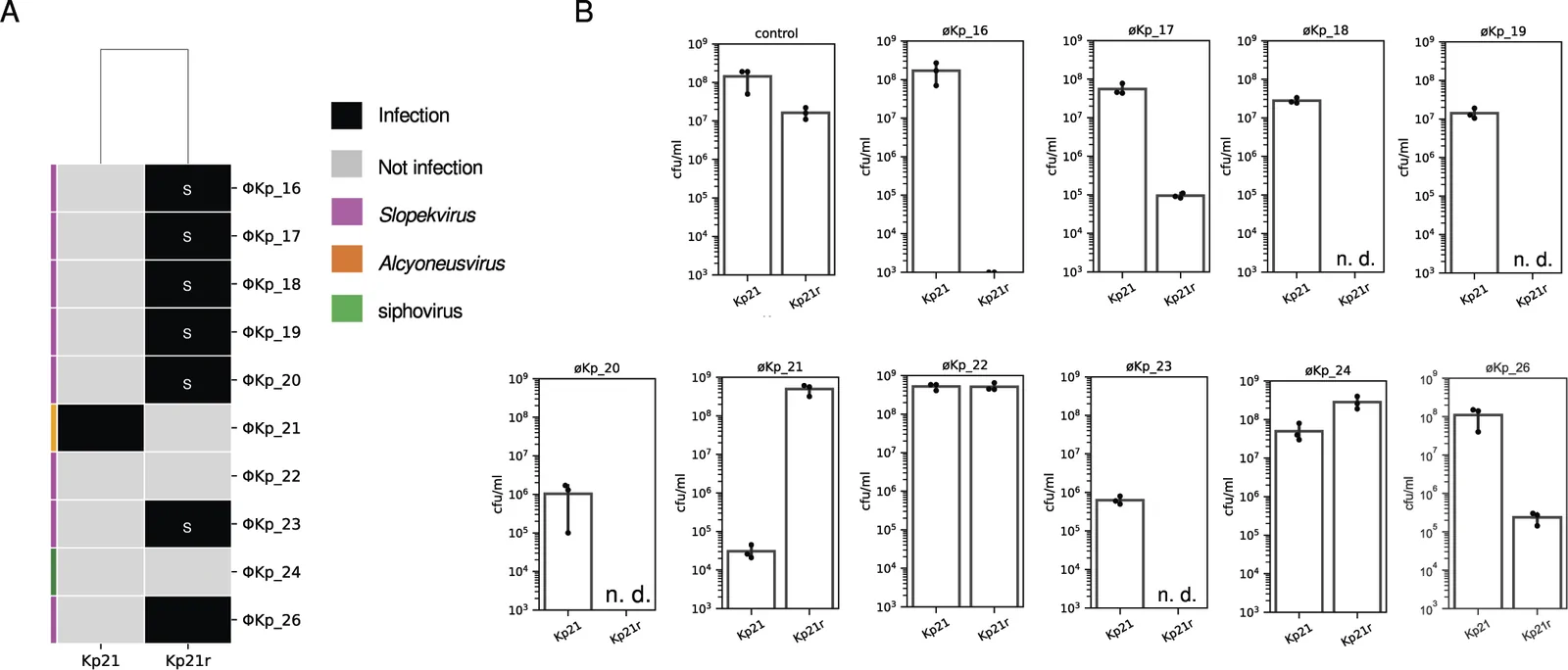
Analysis of the shift in susceptibility to the phage cocktail in Kp21 and Kp21r. (**A**) The host range of Kp21 and Kp21r against a phage cocktail comprising 10 phages. “S” denotes a sparse bacterial lawn. (**B**) Colony-forming units are mentioned under each phage. Kp21 or Kp21r were mixed with individual phages, and 2 h after phage addition, samples were diluted to 10^−2^ and 10^−4^ and lawned onto LB plates. “n.d.” indicates that no colonies were detected at the 10^−2^ dilution.

To investigate the susceptibility of Kp21r to the phage cocktail, we compared phage plaque formation between Kp21 and Kp21r. According to the host range analysis, no phages formed plaques on Kp21. However, the Kp21r strain exhibited susceptibility to øKp_16, 17, 18, 19, 20, 23, and 26, which belong to the *Slopekvirus* genus. This finding suggests that Kp21r becomes susceptible to other phages as compensation for its resistance to øKp21 (Fig. 5A). Furthermore, the Kp21r strain showed a sparse background on Luria-Bertani (LB) agar plates containing *Slopekvirus* strains (Fig. 6A). Importantly, this sparse background was not due to confluent plaque lysis, indicating the presence of a mechanism by which the phage cocktail prevents the emergence of phage-resistant bacteria even without phage infection. To assess the bactericidal efficiency of phages against the Kp21r strain, we measured the colony-forming unit (cfu) values of both Kp21 and Kp21r by individually mixing the phages used in the phage cocktail (Fig. 6B). In the case of øKp_21, the cfu of Kp21 was reduced to 10^4^ cfu/mL, whereas the cfu of Kp21r remained nearly the same as that of the control Kp21r (10^7^ cfu/mL–10^8^ cfu/mL). As for øKp_22 and øKp_24, the cfu per milliliter in Kp21r did not show a considerable decrease compared to that in Kp21, consistent with the observation that these phages are incapable of infecting both Kp21 and Kp21r. However, in other phages, the cfu of the Kp21r strain significantly decreased compared to that of Kp21. Notably, colonies were not detected in combinations of øKp_18, 19, 20, or 23-Kp21r. These results indicate that Kp21r viable cells were effectively eliminated by phages that newly infected Kp21r.

### Characterization of Kp21 and Kp21r

**Fig 7:**
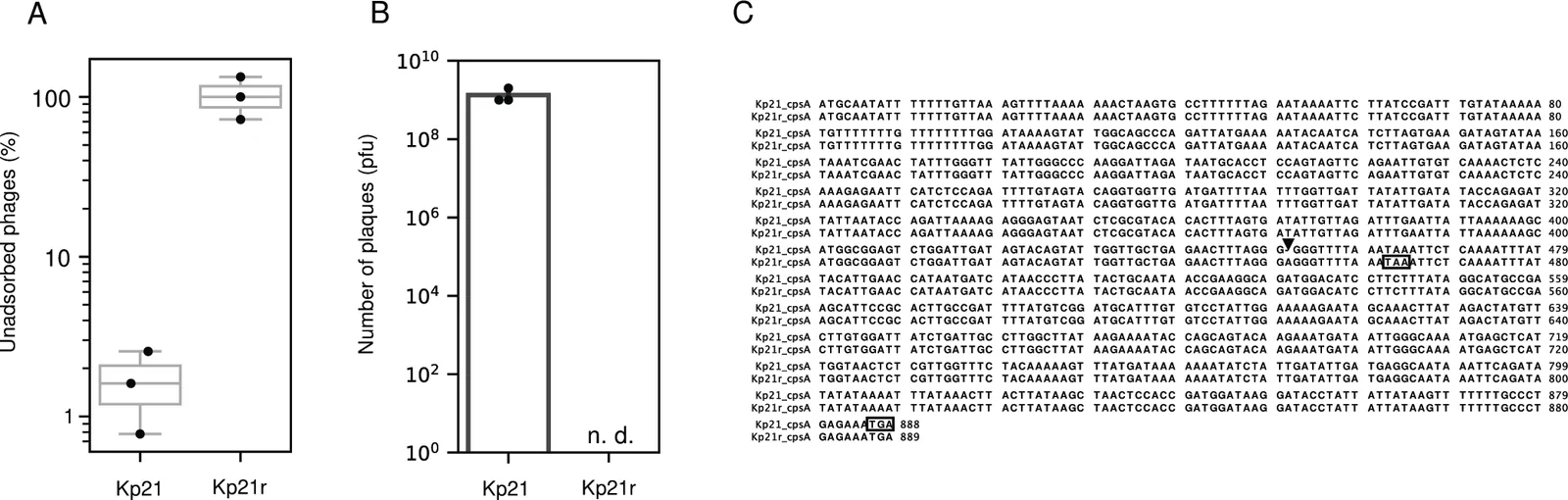
Characterization of the phage-resistant Kp21r strain. (**A**) Adsorption assay of øKp_21 against Kp21 and Kp21r strains. Kp21 and Kp21r were incubated until reaching an OD_600_ of 0.5. Subsequently, øKp_21 was added at an MOI of 0.01, and the mixture was incubated at 37°C with shaking at 200 rpm. After 5 min, 200 µL of the mixture was withdrawn and centrifuged at 9,100 *× g* for 1 min. The number of phages in the supernatant was measured. (**B**) 1.0 × 10^9^ pfu of øKp_21 were mixed with 100 µL of overnight culture of Kp21 or Kp21r. Then, 5 mL of 0.6% YT (yeast extract and tryptone)-soft agar was added to the host and phage mixture and incubated at 37°C overnight. “n.d.” indicates that no plaques were detected. (**C**) Nucleotide sequences of *cpsA* in Kp21 and Kp21r were aligned using ClustalW (https://www.genome.jp/tools-bin/clustalw). The insertion mutation (A) is indicated by an arrow, and the stop codons of *cspA* in Kp21 and Kp21r are shown by a square.

To elucidate the distinctions between the strains Kp21 and Kp21r, we conducted an adsorption assay of øKp_21 for both Kp21 and Kp21r. The assay revealed that the unadsorbed fraction of øKp_21 for the Kp21r strain reached approximately 100% at 5 min after phage addition, whereas for Kp21, this value ranged between 2% and 5% (Fig. 7A). Moreover, no plaques were observed when øKp_21 was added at 1.0 *×* 10^9^ pfu (EOP <10^−9^) (Fig. 7B), indicating that øKp_21 loses its ability to adsorb to Kp21r.

Subsequently, we detected single nucleotide polymorphisms (SNPs) between Kp21 and Kp21r. Insertion mutations were detected in two genes, *thpA* (encoding the inner membrane protein) and *cpsA* (encoding exopolysaccharide synthesis genes), as shown in Table S6. This finding suggests that the øKp_21 phage specifically recognizes the capsular polysaccharide of Kp21. Insertion mutations in *cpsA* in Kp21r were observed at the 452nd nucleotide position, and a stop codon appeared at the 463rd nucleotide position (Fig. 7C). Consequently, 141 amino acids were truncated at the C-terminus of CpsA in Kp21r (154 amino acids long), compared to the wild-type CpsA amino acid with a length of 295 amino acids. This severe truncation likely impairs the biosynthesis of capsular polysaccharide in Kp21r. Additionally, we isolated eight new øKp21-resistant Kp21 strains (Kp21r-1 to Kp21r-8) and sequenced their draft genomes. SNPs and/or insertions/deletions mutations were identified in genes associated with capsular polysaccharide biosynthesis (Table S7). These findings provide further support for our hypothesis that øKp_21 recognizes Kp21 through its interaction with capsular polysaccharide.

## DISCUSSION

Phage therapy is increasingly recognized as an effective strategy for combating antimicrobial-resistant bacteria, especially nosocomial pathogens (8 – 10, 33). A recent study demonstrated the successful suppression of inflammation in a mouse model of inflammatory bowel disease (IBD) through the eradication of *K. pneumoniae* using a phage cocktail, suggesting its potential therapeutic application for treating IBD in humans (34).

In this study, we isolated and characterized newly isolated bacteriophages targeting antimicrobial-resistant *K. pneumoniae* strains harboring the *bla*_IMP-6_ encoding pKPI-6 plasmid. We found 29 phages from sewage samples in western Japan against both *K. pneumoniae* and *E. coli* strains harboring the pKPI-6 plasmid. Genomic sequence analysis revealed that, except for øKp_22, the *Slopekvirus* members in our study shared similar genome sizes and identities, indicating their classification as variants of the same species (Table S2). Given the concerns about the potential presence of antimicrobial resistance (AMR) and virulence factor (VF) genes in our phages, as these genes can be transferred in clinical settings (35 – 37), we conducted an analysis and found no detection of AMR or VF genes in the isolated phage genomes. Consequently, these phages can be safely applied in clinical settings.

Our host range experiment demonstrated that most of the *Slopekvirus* phages formed plaques on the 25 tested *K. pneumoniae* strains. Specific host strains (*K. pneumoniae* Kp12 to Kp20) exhibited higher EOP (Fig. 3) against most phages, suggesting that several host strains exhibit high susceptibility to newly isolated phages isolated from western Japan. Moreover, host range experiment results indicated a positive correlation between EOP and plaque size for several phages, such as øKp_1, 7, 7–1, 14, 24, 27, and 31. To the best of our knowledge, few reports have described the correlation of these factors (38); however, our adsorption assay and one-step growth experiment suggest that plaque size in each host associated with the burst size and/or phage growth rate (Fig. S6). These findings contribute to the development of newly isolated phages with enhanced virulence against the host bacteria and guide the selection of phage strains for the development of phage cocktails (39, 40).

Our SNP analysis suggested that Kp21r exhibits a deficiency in capsular polysaccharide.

Although no differences in colony morphology between Kp21 and Kp21r were observed on LB agar plates or in cell growth in LB medium, it is well known that capsular polysaccharide provides protection against various stressors, including the host immune system and antimicrobial agents (41). Consequently, Kp21r may be more susceptible to such stressors than Kp21 as a trade-off for its resistance to øKp_21.

The phage cocktail experiment revealed that Kp21r acquired susceptibility to other phages in the phage cocktail. In contrast to the results observed with the Kp21 strain, the addition of Kp22 did not impede the emergence of phage-resistant Kp22 (Kp22r). This divergent outcome in the Kp21 and Kp22 cocktail experiments indicates that the phage cocktail is not an all-round strategy. However, to date, it remains the most reliable strategy to combat bacteriophages. The development of a phage cocktail lacks established universal methods or guidelines, making it challenging to predict the most efficient combination of phages to inhibit the emergence of phage-resistant bacteria. Nevertheless, previous studies have suggested that the mixing of several phages that recognize different host receptors is pertinent to effectively reducing the occurrence of phage-resistant bacteria (42 – 44).

Our experimental findings demonstrate that Kp21r exhibited susceptibility to øKp_16, 17, 18, 19, 20, 23, and 26, whereas Kp21 was exclusively susceptible to øKp_21 (Fig. 6). This outcome suggests that phage-resistant bacteria are more vulnerable to attacks by other phages present in the environment during an evolutionary arms race. Adsorption assays revealed that øKp_21 lacks the ability to adsorb to Kp21r. SNP analysis in Kp21 and Kp21r revealed insertion mutations in at least two genes: *cpsA*, which encodes a putative capsular biosynthesis protein, and *thpA*, which encodes a sugar ABC transporter substrate-binding protein. It has been reported that capsular polysaccharides function as the barrier to infect phages (45). In a recent publication, a newly discovered phage that recognizes the capsular polysaccharide of *K. pneumoniae* was reported (46), suggesting that capsular polysaccharide may be a contributing factor enabling øKp_21 to adsorb to its host, Kp21. We observed that the inclusion of *Slopekvirus* in the phage cocktail diminished the lawn density of Kp21r on plates. We posit that this phenomenon was caused by lysis mechanisms such as “lysis from without (LO)” or “rapid lysis” (47, 48). Gp5 in T4 phage, which has tail lysozyme, is known to cause LO (49). Gp5 forms a complex with the T4 phage tail, and when the T4 phage adsorbs to their host, Gp5 degrades the peptidoglycan layer. We found that *Slopekvirus* members, which were used in the phage cocktail in this study, encode a baseplate gene which has a domain of tail lysozyme (Table S8), possessing the capability to degrade peptidoglycan. SNP analysis of Kp21 and Kp21r suggests that Kp21r exhibits deficient capsular polysaccharide, thereby making the phage tail protein encoded in *Slopekvirus* more effective in degrading Kp21r peptidoglycan and leading to rapid lysis. Some studies have reported that phage-resistant bacteria, due to mutations in genes associated with antibiotic resistance, can become susceptible to antibiotics, and combining phages with antibiotics has been shown to effectively eliminate the target bacteria (50 – 53). Our findings demonstrate that combinations of phages and phage-encoded tail lysozyme efficiently eliminate and/or inhibit the growth of phage-resistant growth (54).

In conclusion, we have successfully isolated and characterized newly isolated phages capable of infecting *K. pneumoniae* and *E. coli* harboring the pKPI-6 plasmid, marking a pertinent step toward establishing a public phage bank and advancing phage therapy in Japan. Our phage sets can diminish the threat posed by *K. pneumoniae* harboring the pKPI-6 plasmid in clinical settings. Furthermore, our phage sets encompass an ample variety of phage types suitable for the development of a phage cocktail. However, the development of a high-throughput method is imperative to efficiently isolate and characterize additional new phages.

## MATERIALS AND METHODS

### Phage isolation and host information

A total of 32 IMP-6-producing isolates of *K. pneumoniae* and one IMP-6-producing isolate of *E. coli* were isolated from clinical settings in western Japan. Additionally, 29 newly isolated phages were collected from sewage in western Japan. To begin, 100 µL of sewage was mixed with an overnight culture of the indicator host, and this mixture was then added to 3 mL of 0.6% YT-soft agar prior to inoculation onto Luria-Bertani (LB) agar plates. The plates were subsequently incubated at 37°C overnight; after which, single-plaque isolation was performed. The plaques were suspended in 1 mL of LB medium and incubated for 2 h. Next, 50 µL of chloroform (FUJIFILM Wako Pure Chemical Corporation, Osaka, Japan) was added to each solution, and the mixture was vortexed before being centrifuged at 10,000 *× g* for 10 min at 4°C. The supernatants were then mixed with individual indicator hosts and incubated at 37°C overnight on LB agar plates. The single-plaque isolation procedure was repeated three times, and the isolated phages were stored at 4°C until further use. Kp21 was renamed from the *K. pneumoniae* f22 strain.

### Phage propagation and purification

Pre-cultured host strains were inoculated into 3 mL of fresh LB medium (diluted 1:100) and incubated at 37°C until reaching an OD_600_ of 0.5. Subsequently, each phage, originally isolated using the indicated host, was added to the culture and incubated at 37°C with shaking at 200 rpm for 4–6 h. Following the lysis, 50 µL of chloroform (FUJIFILM Wako Pure Chemical Corporation) was added to 1 mL of the phage lysate, vortexed, and then centrifuged at 9,100 *× g* for 10 min. The resulting supernatants were filtered through a 0.22 µm pore-size membrane (Millipore, MA, USA). Cesium chloride (CsCl) density gradient phage purification was performed as described previously (55, 56) with some modifications. Specifically, 10% polyethylene glycol 6000 (FUJIFILM Wako Pure Chemical Corporation) and 0.5 M NaCl were added to the phage lysates and kept at 4°C for 1.5 h. Following this, the phage lysates were centrifuged at 10,000 × *g* for 30 min, and the resulting phage pellets were suspended in 1 mL of TM buffer (10 mM Tris-HCl and 5 mM MgCl_2_ [pH 7.5]). Subsequently, 100 µg/mL of DNase I (Roche, Basel, Switzerland) and RNase I (Thermo Fisher Scientific, MA, USA) were added to the phage solution and incubated at 37°C for 30 min. CsCl (FUJIFILM Wako Pure Chemical Corporation) at three different densities (*ρ* = 1.3, 1.5, and 1.7) and the phage solution were layered in tubes and ultracentrifuged (Optima MAX-TL; Beckman Coulter, California, USA) at 100,000 *× g* for 1 h. The resulting phage bands were then collected and dialyzed in SM buffer (25 mM Tris-HCl [pH 7.5], 100 mM NaCl, and 8 mM MgSO_4_).

### Phage propagation and electron microscopic imaging

Copper mesh grids coated with formvar and carbon (Veco grids; Nissin EM, Tokyo, Japan) were glow-discharged and then placed on drops of the phage suspension for 1 min. Subsequently, the grids were rinsed with distilled water and stained with a 2% uranyl acetate solution. The samples were examined using transmission electron microscopy (HT7700; Hitachi Ltd., Tokyo, Japan) at 80 kV.

### Host range determination and EOP assay

Each host was incubated at 37°C overnight, and 100 µL of each overnight culture was mixed with 100 µL of each phage suspension. Next, 5 mL of 0.6% YT-soft agar was added to the host-phage mixture and inoculated onto LB agar plates. The plates were then incubated at 37°C overnight, and the number of plaques on each plate was counted. The EOP was calculated using the formula below:


EOP=numberofplaquesfromtheindividualphage−hostcombination/numberofplaquesfromtheindicatorhost−phagecombination.

The detection limit for EOP was set at 10^−4^ pfu. EOP values were measured for all phage-bacteria combinations. Each plaque image was captured using a scan1200 (Interscience, Montpellier, France) for measuring their sizes. The size of each plaque size (mm^2^) was measured using Fiji (https://fiji.sc) version 2.3.0, with a conversion factor of 11 pixels per millimeter. For very small plaques, the edge of individual plaques was detected using the “find edge” tool in Fiji. Ten plaque areas were measured for each phage-host combination if the number of plaques on the plate exceeded 10.

### OD_600_ kinetics and cocktail experiment

The host colony was pre-cultured in LB medium overnight at 37°C. Subsequently, the pre-cultured bacteria were inoculated (1:100) into fresh LB medium and incubated at 37°C with shaking at 200 rpm until reaching an OD_600_ of 0.1. Each indicated phage was added to the culture at a concentration of 1.0 × 10^8^ pfu, and the mixed culture was incubated at 37°C with shaking at 200 rpm. The OD_600_ was measured at appropriate time intervals over a 24-h period. In the phage cocktail experiments, a combination of 10 phages (øKp_16, 17, 18, 19, 20, 21, 22, 23, 24, and 26) was mixed at a concentration of 1.0 × 10^7^ pfu/mL for each individual phage. All experiments in this section were performed in triplicate.

### Host and phage genome sequences

All phage genomic DNA was extracted using the Norgen phage DNA isolation kit (Norgen Biotak, Birmingham, UK), following the manufacturer’s instructions. Each phage DNA library was constructed using the QIA seq FX DNA library kit (Qiagen), and sequencing was performed on the Illumina MiSeq platform. Genome assembly was performed using Shovill with default settings. Phage contigs were filtered based on a contig length <200 and coverage of <25. Bacteria and phage strains were annotated using prokka (57) or PGAP (58) version 2021-07-01.build5508. For Nanopore long-read sequencing, we used the Monarch HMW DNA Extraction Kit for Tissue (NEB, MA, USA) following the manufacturer’s instructions.

A long-read library was prepared using the Rapid Barcoding kit (Oxford Nanopore Technologies, Oxford, United Kingdom, catalog number: SQK-RBK004) and sequenced on an R9 flow cell (Oxford Nanopore Technologies, catalog number: FLO-MIN106) using a GridION device (Oxford Nanopore Technologies). Basecalling was performed using Guppy version 5.0.12 in high accuracy mode. The obtained long reads, as well as the MiSeq short reads, which were trimmed using fastp v0.20.1, were assembled using Unicycler v0.4.8 with default parameters. Annotation was conducted using PGAP version 2021-07-01.build5508.

### Bioinformatics analysis

For protein prediction in phages, we constructed the phage protein databases from the International Committee on Taxonomy of Viruses (ICTV), which comprise 4,312 genomes and 4,62,579 proteins (https://www.ncbi.nlm.nih.gov/genomes/GenomesGroup.cgi?taxid=28883) (September 2021), using local blastp (59) with an *e*-value threshold of <2e−20. Protein domains were identified using hmmer (https://www.ebi.ac.uk/Tools/hmmer/) with the Pfam-A 35.0 database, employing an *e*-value of <1e−10. Phage classification was determined based on the NCBI GenBank and ICTV. The average nucleotide identity was calculated using the average_nucleotide_identity.py program in the pyani packages (60). MUMmer was used to align nucleotide sequences. AMR genes and virulence genes were detected using ABRicate version 1.0.1 (https://github.com/tseemann/abricate) under default settings. The ResFinder database was used to extract AMR genes (61), and the Virulence Factors Database was used to extract virulence genes (62). The packaging mechanism and terminal repeats were analyzed using Phagetermvirome version 4.0.1 (63), and tRNAs were detected using tRNAscan-SE 2.0 (64). Kp21 and Kp21r SNP analysis was performed using SNIPPY (65) with default settings. The anti-defense system in each phage was predicted using AcrFinder (66), employing default settings.Each sequence type and serotype based on the K locus were determined using Kleborate (67). Each phage lifecycle was presumed using PhaTYP (68).

### Taxonomic classification

Taxonomy classification was performed using vConTACT2 v0.11.3 with the Prokaryotic Viral RefSeq211-Merged database, under default settings (19). The resulting network was visualized using cytoscape. Each proposed taxonomy was further validated using the ICTV 2022 taxonomy classification. All phage classifications are available in Table S2. ANI was conducted using the average_nucleotide_identity.py program in pyani packages (60).

### Phage-resistant Kp21 (Kp21r) and Kp22 (Kp22r) strains

Kp21 and Kp22 were cultured with øKp_21 or øKp_22, respectively, at 37°C. After 24 h of incubation, 1 mL of each culture was centrifuged at 4,400 *× g* for 10 min. The supernatant was discarded, and the pellets were washed with LB medium. This washing procedure was repeated twice. Subsequently, the pellet was resuspended in saline solution (0.85% NaCl), and the suspension was plated on LB agar and incubated at 37°C overnight. A single colony was selected and incubated overnight at 37°C with shaking at 200 rpm. The glycerol stock of the Kp21r culture was stored at −80°C until further use.

### Kp21 and Kp21r adsorption assay

Kp21 and Kp21r were cultured in LB medium and incubated at 37°C until reaching an OD_600_ of 0.5. Subsequently, 3.0 × 10^6^ pfu of øKp21 was added (MOI = 0.01) and incubated at 37°C with shaking at 200 rpm for 5 min. Next, 20 µL of chloroform was added to 200 µL of the mixture and vortexed. The samples were then centrifuged at 9,100 *× g* for 1 min, and the supernatant was collected. A 100 µL aliquot of the supernatant was mixed with Kp21, and plaque assays were performed to determine the number of unadsorbed phages. The percentage of unadsorbed phages was calculated using the formula below:


unadsorbedphage%=(numberofphagesinthesupernatant/addedphages)×100

### Characterization of switched phage sensitivity between Kp21 and Kp21r

The phage sensitivity of Kp21 and Kp21r was examined. Briefly, 1.0 × 10^9^ pfu of each phage included in the phage cocktail were mixed with 100 µL of overnight Kp21 or Kp21r culture. Subsequently, 5 mL of 0.6% YT-soft agar was added to the host-phage mixture and then poured onto LB agar and incubated at 37°C overnight. The colony count was examined as follows: Kp21 and Kp21r were incubated at 37°C until reaching an OD_600_ of 0.1. Then, each phage was added at 1.0 × 10^9^ pfu, and the mixture was incubated at 37°C. After 2 h of incubation following phage addition, the mixture was collected and centrifuged at 3,300 *× g* for 15 min. The supernatant was discarded, and the pellet was suspended in 500 µL of phosphate-buffered saline (0.137 M NaCl, 0.27 mM KCl, 0.1 M Na_2_HPO_4_, 18 mM KH_2_PO_4_). The suspension was diluted to 10^−2^ and 10^−4^, and 100 µL of each dilution was spread onto LB agar.

### One-step growth experiment

Host *K. pneumoniae* was grown overnight at 37°C. The overnight culture was diluted 1:100 in LB medium and incubated until the OD_600_ reached 0.5. Each phage was added at an MOI of 0.25 or 0.5 against each host strain and incubated in the water bath for 6 min at 37°C for phage adsorption to the host. Then, 100 µL of the culture was withdrawn and centrifuged at 13,000 × *g* for 2 min, and the supernatant was transferred to 96-well tubes. Additionally, 1 µL of the culture was transferred to 10 mL of fresh LB medium. Samples were withdrawn every 8 min, and 100 µL of each sample was centrifuged at 13,000 × *g* for 1 min, and the supernatant was transferred to 96-well plates. Tenfold dilutions were spotted on 0.6% LB-soft agar containing each host showing a higher EOP. The burst size was calculated using the formula below:


burstsize=(numberofproducedphagesattheplateau−numberofphagesat0minutes)/(numberofinfectedcells)

### Statistical analysis

All statistical analyses were conducted using a two-sided Student *t* test with Python version 3.9.7 and the SciPy Module version 1.4.1. A *P-*value* *less than 0.05 was considered statistically significant.

## SUPPLEMENTAL MATERIAL

Supplemental Figures spectrum.04761-22-s0001.pdf Fig. S1 to Fig. S6.

Supplemental Tables spectrum.04761-22-s0002.xlsx Tables S1 to S8.

ASM does not own the copyrights to Supplemental Material that may be linked to, or accessed through, an article. The authors have granted ASM a non-exclusive, world-wide license to publish the Supplemental Material files. Please contact the corresponding author directly for reuse.
