# Supplementary material for: Characterization of 29 newly isolated bacteriophages as a potential therapeutic agent against IMP-6-producing Klebsiella pneumoniae from clinical specimens
Source: Microbiol Spectr. 2023 Sep 19;11(5):e04761-22. doi: 10.1128/spectrum.04761-22 (PMC10581060; doi:10.1128/spectrum.04761-22)
Supplement: Supplemental Figures — Fig. S1 to Fig. S6. [file spectrum.04761-22-s0001.pdf]

Fig. S1

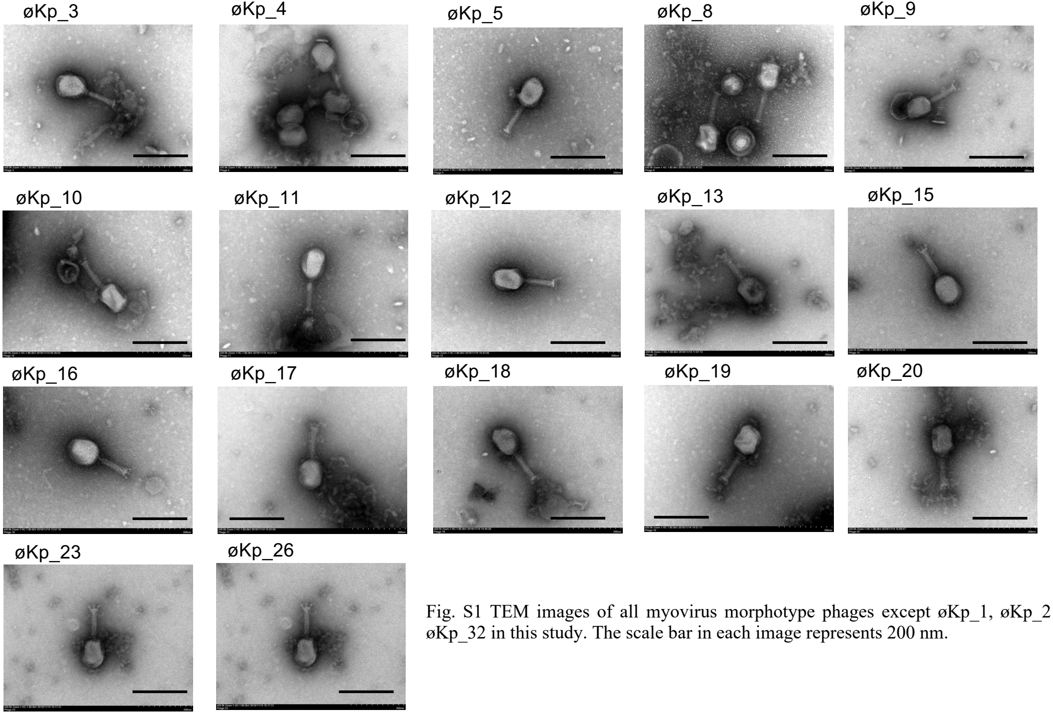

Fig. S1 TEM images of all myovirus morphotype phages except øKp_1, øKp_21, øKp_22 and øKp_32 in this study. The scale bar in each image represents 200 nm.

Fig. S2A

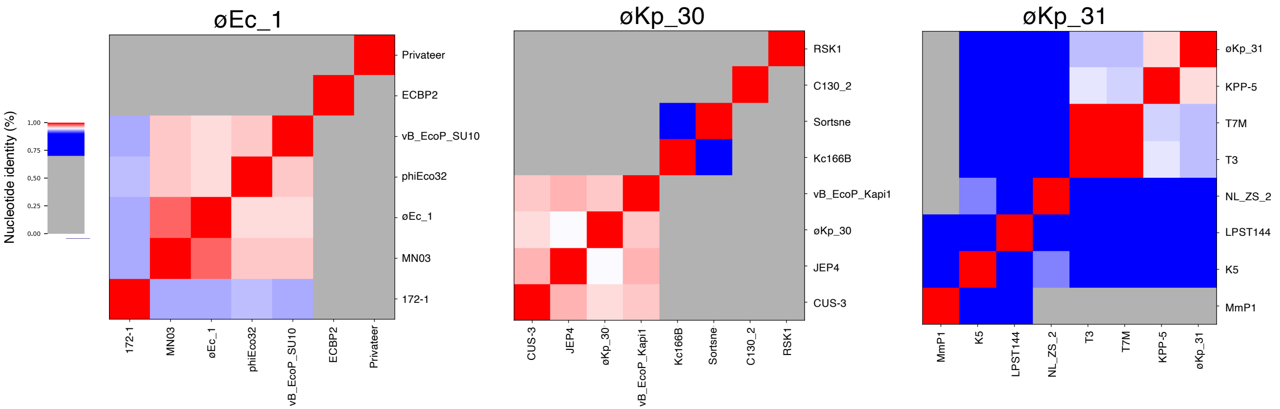

Fig. S2 Average nucleotide identity was conducted using the average_nucleotide_identity.py program in the pyani packages (60). (A) Nucleotide identity of the podovirus group (A), siphovirus group (B), and myovirus group (C)

Fig. S2B

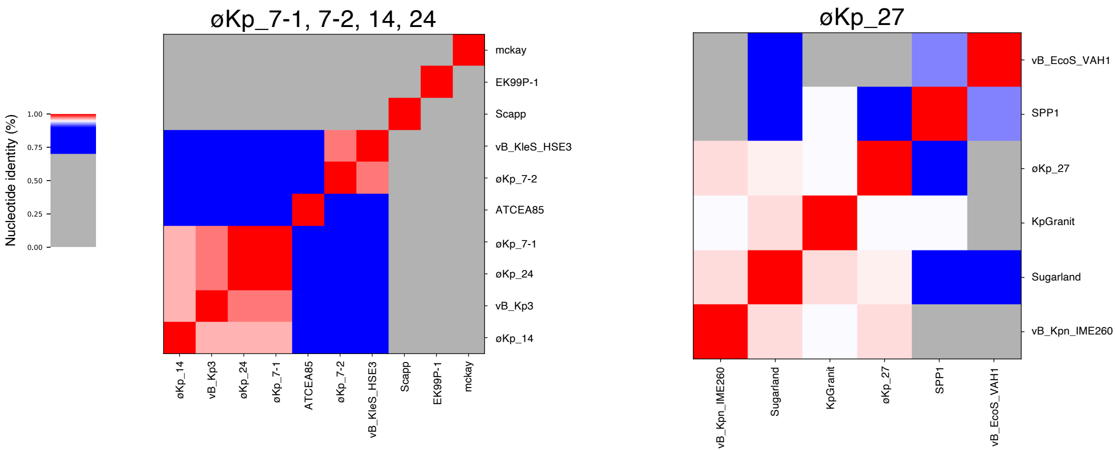

Fig. S2 Average nucleotide identity was conducted using the average_nucleotide_identity.py program in the pyani packages (60). (A) Nucleotide identity of the podovirus group (A), siphovirus group (B), and myovirus group (C)

Fig. S2C

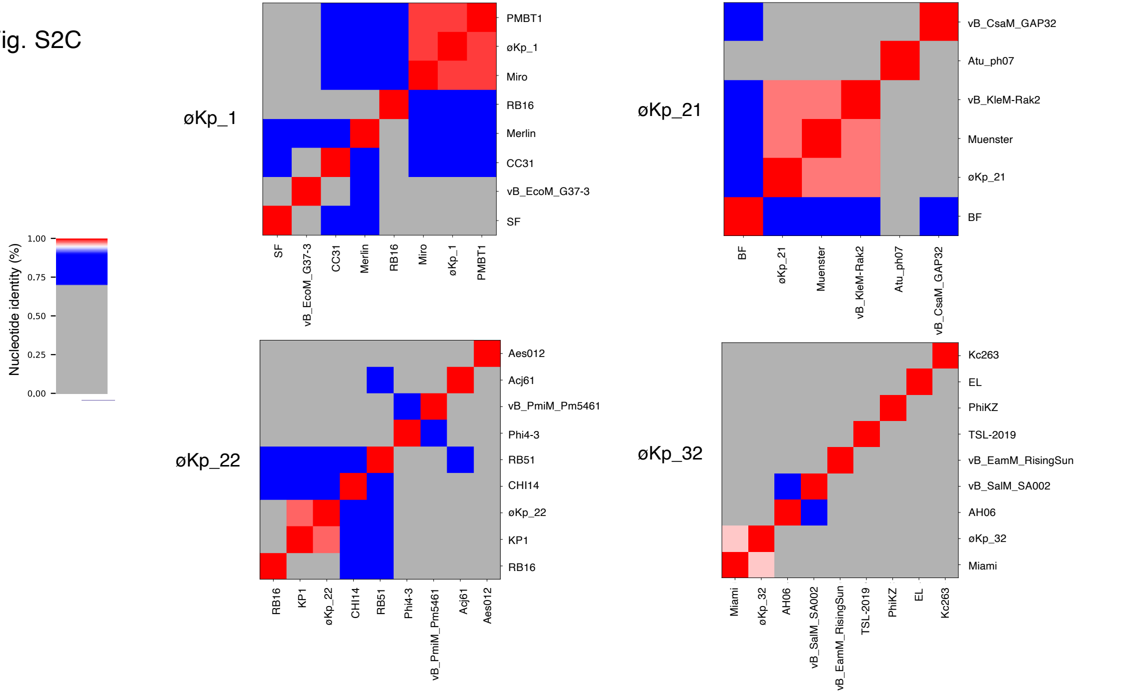

Fig. S2 Average nucleotide identity was conducted using the average_nucleotide_identity.py program in the pyani packages (60). (A) Nucleotide identity of the podovirus group (A), siphovirus group (B), and myovirus group (C)

Fig. S3

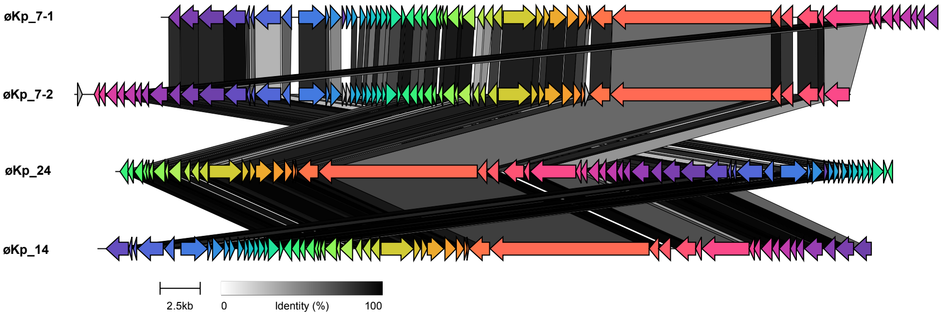

Fig. S3 Genome comparison of four unclassified siphoviruses.

Fig. S4

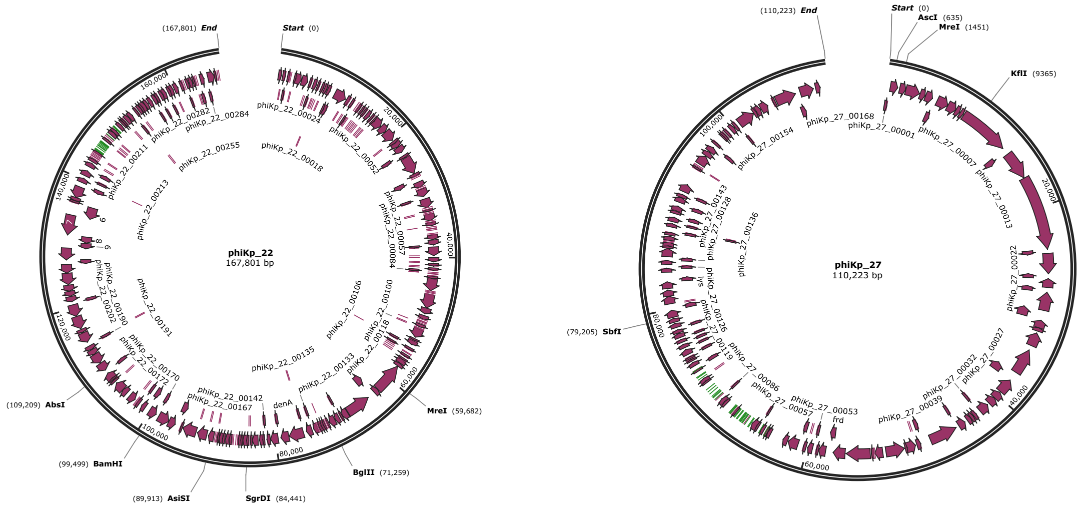

Fig. S4 Genome map of øKp_22 and øKp_27. These two phages encode a considerable number of tRNAs (16 tRNA and 27 tRNA, respectively.) in this study. The encoded tRNAs in each phage are highlighted in green.

Fig. S5

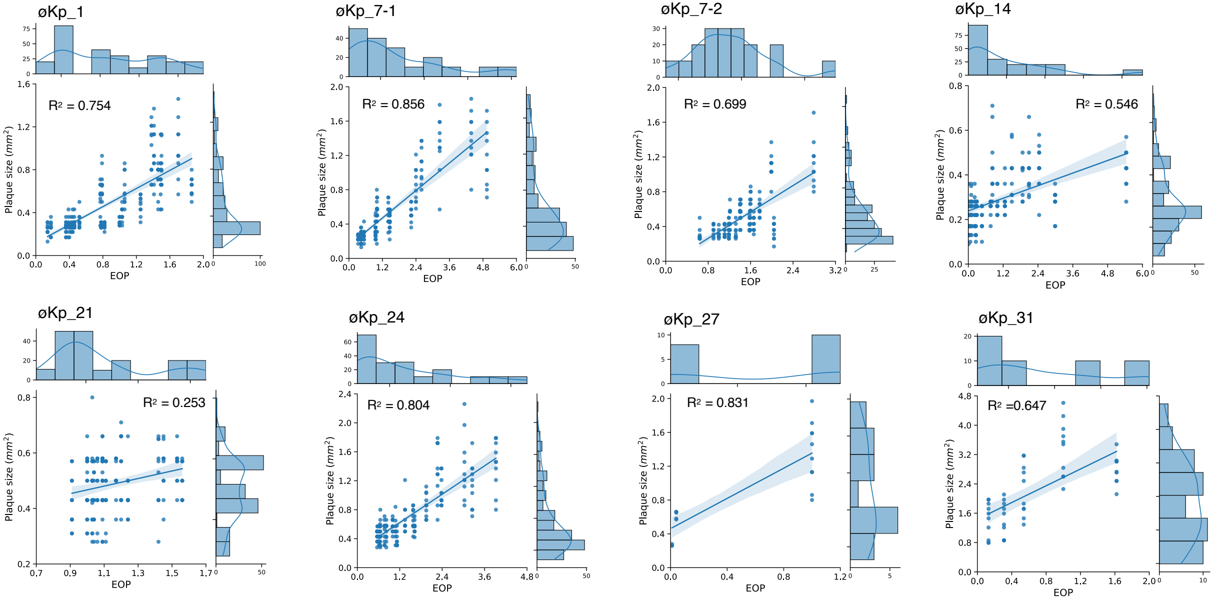

Fig. S5 Correlation between plaque size and EOP. Plaque size (mm^2) and EOP were measured in phages from representative phage strains. The X- and Y-axes represent the EOP in each phage and the plaque size, respectively. A maximum of 10 plaques were randomly selected in individual phage-host combinations, and plaque sizes were measured using ImageJ. Python packaging Seaborn was used to visualize the correlation, and the R correlation in each combination was calculated using Scipy version 1. 8. 1.

Fig. S6

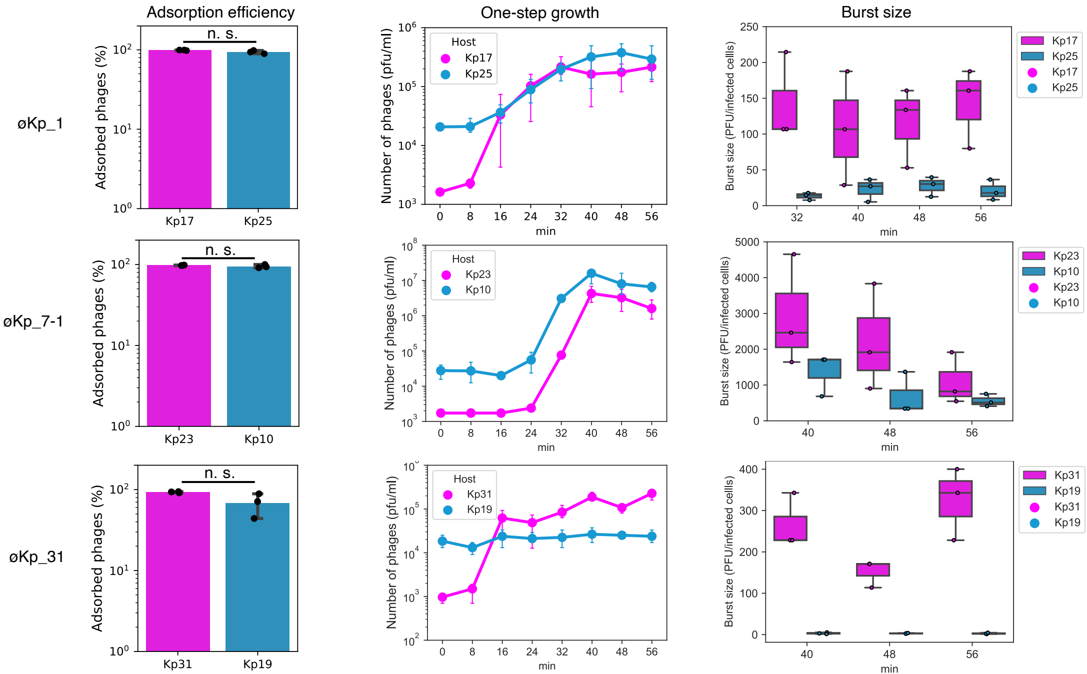

Fig. S6 The one-step growth analysis. We selected three different phages (øKp_1, øKp_7-1, and øKp_31) and two hosts showing higher and lower EOP for each phage. Each phage was added at an MOI of 0.25 or 0.5 against each host strain based on their EOP. The adsorption efficiency was calculated by subtracting the number of unadsorbed phages from the total number of phages. The culture was diluted (10^4 dilution) into fresh LB medium, and samples were withdrawn at 8-min intervals until 56 min had elapsed. The number at phages in each time point was counted using the host showing high EOP. “n. s.” indicates that adsorption rate was not significant. $P < 0.05$ was considered as significant difference.
